# Sudden Cardiac Death Risk in Downhill Skiers and Mountain Hikers and Specific Prevention Strategies

**DOI:** 10.3390/ijerph18041621

**Published:** 2021-02-08

**Authors:** Josef Niebauer, Martin Burtscher

**Affiliations:** 1 University Institute of Sports Medicine, Prevention and Rehabilitation, Paracelsus Medical University, Lindhofstraße 20, 5020 Salzburg, Austria; 2 Ludwig Boltzmann Institute for Digital Health and Prevention, Lindhofstraße 22, 5020 Salzburg, Austria; 3 Department of Sport Science, University of Innsbruck, A-6020 Innsbruck, Austria; martin.burtscher@uibk.ac.at

**Keywords:** mountains, exercise, altitude, fitness, cardiovascular, metabolic

## Abstract

Sudden cardiac death (SCD) still represents an unanticipated and catastrophic event eliciting from cardiac causes. SCD is the leading cause of non-traumatic deaths during downhill skiing and mountain hiking, related to the fact that these sports are very popular among elderly people. Annually, more than 40 million downhill skiers and mountain hikers/climbers visit mountainous regions of the Alps, including an increasing number of individuals with pre-existing chronic diseases. Data sets from two previously published case-control studies have been used to draw comparisons between the SCD risk of skiers and hikers. Data of interest included demographic variables, cardiovascular risk factors, medical history, physical activity, and additional symptoms and circumstances of sudden death for cases. To establish a potential connection between the SCD risk and sport-specific physical strain, data on cardiorespiratory responses to downhill skiing and mountain hiking, assessed in middle-aged men and women, have been included. It was demonstrated that previous myocardial infarction (MI) (odds ratio; 95% CI: 92.8; 22.8–379.1; *p* < 0.001) and systemic hypertension (9.0; 4.0–20.6; *p* < 0.001) were predominant risk factors for SCD in skiers, but previous MI (10.9; 3.8–30.9; *p* < 0.001) and metabolic disorders like hypercholesterolemia (3.4; 2.2–5.2; *p* < 0.001) and diabetes (7.4; 1.6–34.3; *p* < 0.001) in hikers. More weekly high-intensity exercise was protective in skiers (0.17; 0.04–0.74; *p* = 0.02), while larger amounts of mountain sports activities per year were protective in hikers (0.23; 0.1–0.4; <0.001). In conclusion, previous MI history represents the most important risk factor for SCD in recreational skiers and hikers as well, and adaptation to high-intensity exercise is especially important to prevent SCD in skiers. Moreover, the presented differences in risk factor patterns for SCDs and discussed requirements for physical fitness in skiers and hikers will help physicians to provide specifically targeted advice.

## 1. Introduction

Mountain sport activities are becoming more and more popular all over the world. Annually, more than 10 million tourists are attracted by the mountainous areas of Austria alone, 40 million for the Alps in total and 100 million worldwide [1]. Whereas participation in regular sports activities is associated with considerable health benefits, especially vigorous exercise in unaccustomed individuals may sever as trigger for injury, illness or even sudden death [2,3]. Large data sets derived from the general population convincingly indicate an almost linear relationship between increasing cardio-respiratory fitness (CRF) and longevity [4]. The risk–benefit ratio, however, depends on the risks associated with the specific sport activity. Mountain sports possess inherent risks due to objective hazards like rock and ice falls, avalanches, lightnings, etc. but also related to subjective reasons like inflated self-esteem, insufficient sport-specific skills and/or physical fitness [5,6]. For instance, the level of individual physical fitness needs to be much higher for uphill walking in the mountains, often with heavy boots and additional load (rucksack), compared to level hiking [7]. Moreover, extreme environmental conditions, i.e., cold, heat, wind, rain and snowfall, hypoxia/altitude, may aggravate individual cardiorespiratory stress [8]. Considering the large number of mountain hikers and downhill skiers with their broad range of age and fitness levels, the occurrence of sudden cardiac death (SCD) is not unexpected. However, the large proportion of SCDs contributing to the total number of deaths during mountain sports is somewhat surprising [9]. Indeed, 50% of all deaths during downhill skiing and mountain hiking are SCDs, unfavorably impacting the risk-benefit ratio of these activities [5,6]. Appropriate preventive measures are urgently needed to reduce risk of SCD and to optimize beneficial effects of exercise when skiing and/or hiking in the mountains. Available guidelines on general prevention are helpful but should be complemented by recommendations specifically focusing on aspects related to the type of sport, i.e., downhill skiing or mountain hiking. Therefore, here, we compare risk factors and risk conditions between those sports in order to derive specific suggestions for SCD prophylaxis in skiers and hikers at risk.

## 2. Materials and Methods

For comparison of the SCD risk between downhill skiers and mountain hikers, data sets from 2 case-control studies previously published were compared [10,11]. In brief, all deaths that occurred over a nine-year observation period during alpine sport activities in Austria were recorded by members of the Ministry of the Interior. SCD was defined as unexpected, non-traumatic death in persons with or without pre-existing disease who died within 1 h of the onset of symptoms [10]. As more than 90% of all SCDs during downhill skiing and mountain hiking affected males aged over 34, this population constituted the cases [9]. With regard to skiers suffering from SCD, out of a total of 157 cases, 104 fulfilled the inclusion criteria (meeting SCD definition, Austrian or German nationality, male sex and age >34 years), and regarding hikers suffering from SCD, from a total of 361 cases 301 fulfilled the inclusion criteria. Further data collection was performed by the use of standardized questionnaires and telephone interviews with close relatives of victims. Data of interest included demographic variables, cardiovascular risk factors, medical history, physical activity, and additionally symptoms and circumstances of sudden death for cases. Mild to moderate habitual physical activity was defined as needing up to 5 metabolic equivalents (METs; 1 MET = 3.5 mL/kg/min oxygen uptake) and strenuous activities of 6 or more METs. Finally, completed questionnaires and telephone interviews included 68 skiers and 179 hikers with SCD. Controls were recruited from skiers (*n* = 720) and hikers (*n* = 960) in the same regions where SCDs occurred and the same data were collected as for the cases. Finally, controls were matched to the cases in terms of age, nationality, type and frequency of mountain sports activities. A total of 204 controls were matched to 68 skiers with SCD, and 537 controls to 179 hikers with SCD (Table 1). Both studies were conducted in accordance with the Declaration of Helsinki 1975. Informed consent was obtained from all subjects involved in the studies.

### Statistics

Data from the epidemiological studies (case-control analyses) are mainly presented as frequencies. Differences in cardiovascular risk factors, demographic characteristics and physical activity data were evaluated by Mann–Whitney, Chi-square or Fisher‘s exact tests and logistic regression analysis was applied for the estimation of adjusted odds ratios (and 95% confidence intervals) for cardiac death outcome.

A *p*-value < 0.05 was considered statistically significant.

## 3. Results

### SCD Risk Factor Differences between Skiers and Hikers

About 50% of all SCDs recorded occurred on the first day of downhill skiing or mountain hiking. SCDs were most frequently observed in the late morning and accumulated with increasing duration from the last food and fluid intake, without differences between activities [10,11]. Significantly different risk factor ratios (SCD victims vs. controls) between skiers and hikers were found for prior myocardial infarction, hypercholesterolemia and physical activity habits (Table 1). Whereas prior myocardial infarction was associated with an increased risk of SCD in skiers, it was hypercholesterolemia in hikers. Additionally, a history of less engagement in light to moderate physical activity and mountain sports activities was associated with a higher risk for SCD in mountain hikers, and skiers who became SCD victims performed high-intensity exercise less often.

Adjusted logistic regression analysis revealed that downhill skiers with a previous MI had a 92.9 (22.8–379.1) times higher adjusted SCD risk and skiers with hypertension a 9.0 (4.0–20.6) fold increased risk (Table 2). In skiers who performed high-intensity exercise more than once per week, the SCD risk was reduced by 83% (0.17; 0.04–0.74). Mountain hikers with a previous MI had a 10.9 (3.8–30.9) times higher adjusted SCD risk and hikers with diabetes a 7.4 (1.6–34.3) fold increased risk. Those hikers who performed mountain sports activities for more than 2 weeks per year benefited from a 77% risk reduction (0.23; 0.1–0.4) (Table 2).

## 4. Discussion

Main findings are that recreational skiers and hikers with previous MI history were most likely to suffer from SCD during their sport activities. Therefore, public health care providers as well as the physicians should alert especially this population to take measures in order to prevent SCD. Moreover, especially skiers should engage in high-intensity exercise at least twice per week to prevent SCD.

Lifestyle characteristics and associated risk factors may differ between populations at risk performing different type of sports, i.e., downhill skiing or mountain hiking [12].

### 4.1. Cardiovascular and Metabolic Effects Related to the Type of Work/Exercise

Downhill skiing is primarily characterized by performing negative work (eccentric exercise) while positive work (concentric exercise) is done when mountain hiking uphill and negative work when walking downhill [13]. We here only consider uphill walking as it is associated with much higher cardiovascular stress then walking downhill [14]. Depending on the skiing skills, technique, skiing speed and terrain, downhill skiing includes also isometric and even concentric exercise [15]. Thus, cardiovascular, respiratory and metabolic responses may considerably differ between individuals. Commonly, several interval-runs of about 1 to 2 min are performed interspersed by short recovery periods and longer resting periods during the passive ascent by lift or cable car. In fact, we have previously reported that during downhill skiing the active skiing time was 44 ± 5% [16]. The cardiovascular stress results from the intensity and the duration of skiing activity and is related to the individual level of fitness [15]. In contrast, mountain hiking (uphill) is performed continuously up to several hours and the cardiovascular stress primarily depends on the velocity of ascent related to the individual fitness.

Beside other lifestyle factors, the type of exercise performed (positive work during uphill hiking and negative work during downhill skiing) might differently impact metabolic factors. For instance, it was demonstrated that uphill walking primarily resulted in reduced triglyceride levels while downhill walking was associated with improved glucose tolerance [17]. Although we are not aware of any direct comparisons between skiers and hikers, we also observed several beneficial metabolic and vascular effects in healthy, elderly downhill skiers that comprised a significant decrease in body fat mass, total cholesterol, low-density lipoprotein (LDL), homocysteine, an improvement in glucose homeostasis, insulin sensitivity, endothelial progenitor cells and peripheral vascular tone as well as exercise capacity [18,19,20]. Therefore, also alpine skiing, when performed regularly, can exert anti-atherogenic effects, that are otherwise not seen in those who only infrequently engage in or even die while performing this type of sport.

Of note, about 50% of all SCDs occurred on the first day of hiking or skiing and were most frequently observed in the late morning hours and were thus associated with increased duration since the last food and fluid intake. These observations clearly point to physiological stress factors like unaccustomed physical activity, mental exposure, dehydration and depletion of carbohydrate stores which may all contribute to elevated sympatho-adrenergic activity, finally triggering harmful cardiovascular responses like arrhythmias and elevated systemic blood pressure [3,21]. In addition, extreme ambient temperatures and hypoxia at high altitude may be triggers for SCD [22,23]. Additionally, pulmonary hypertension during acute exposure to high altitude was suggested to provoke left ventricular dysfunction in individuals suffering from heart disease [24].

### 4.2. Prevention of SCD in Downhill Skiers and Mountain Hikers

Appropriate physical fitness and regular exercise training are essential preventive measures for all. Both the least and optimal amount and intensity of exercise has most recently been reiterated by the World Health Organization and the European Society of Cardiology which call for physical exercise training of 3–7 days per week, totaling 150–300 min of moderate-intensity or 75–150 min of vigorous-intensity aerobic exercise or an equivalent combination thereof per week [25,26]. In subjects 35 years and older who wish to engage in competitive sports or who exercise at a comparable intensity and volume, cardiovascular evaluation is recommended and should include as a minimum family history, symptoms, physical examination, and 12-lead resting ECG. Clinical evaluation, including maximal exercise testing, should be considered for prognostic purposes in sedentary people and individuals with high or very high cardiovascular risk who intend to engage in intensive exercise programs or competitive sports. In selected individuals without known coronary artery disease (CAD) who have very high cardiovascular disease (CVD) risk (e.g., SCORE > 10%, strong family history, or familial hypercholesterolemia) and want to engage in high- or very high-intensity exercise, risk assessment with a functional imaging test, coronary computed tomography angiography (CCTA), or carotid or femoral artery ultrasound imaging may be considered [26].

Individuals with the cardiac risk factors obesity, arterial hypertension, dyslipidemia, or diabetes should also perform resistance training ≥3 times per week to improve hemoglycemic control and insulin sensitivity [27]. If stable coronary artery disease is present, risk stratification is recommended with the possible outcome that skiing and/or mountain hiking may have to be postponed until medical tests have been completed and an adequate physical fitness has been achieved [28]. Once a patient has been cleared for mountainous exercises, rest or only slight physical activity on the first one or two days in the mountains and a gradual increase in activity on the following days should be pursued. These prophylactic measures should be complemented by repeated rests with energy and fluid intake, i.e., every 30 to 60 min, during skiing and hiking activities as well. Sleeping at somewhat higher altitude before exercising on the first day in the mountains may provide some protection, possibly due to short-term acclimatization or a kind of hypoxia preconditioning [23].

High altitude, which may start as low as 1500 m above sea level, is associated with an increase in systolic and diastolic blood pressure and heart rate. In patients on chronic medication, individual treatment should be continued and modified according to documented effects of altitude on these parameters [28].

## 5. Conclusions

In conclusion, previous MI history represents the most important risk factor for SCD in both recreational skiers and hikers, and adaptation to high-intensity exercise is especially important to prevent SCD in skiers. Moreover, the presented differences in risk factor patterns for SCDs and discussed requirements for physical fitness in skiers and hikers will help physicians to provide specifically targeted advice.

## Figures and Tables

**Table 1:** Comparison of characteristics, risk factors and physical activity habits between downhill skiers and hikers with SCD and Controls.

Study Population	Skiers			Hikers			*p*-Value ^a^
	with SCD	Control	*p*-Value	with SCD	Control	*p*-Value	
Number	68	204		179	532		
**Characteristics**							
Age, years	58.6 (±10.5)	58.5 (±8.3)		60.6 (±9.0)	59.4 (±8.2)		
Height, cm	176.2 (±6.5)	177.0 (±6.9)		175.0 (±6.3)	176.9 (±18.5)		
Body mass, kg	79.0 (±10.5)	81.1 (±10.9)		79.1 (±10.6)	76.3 (±8.4)		
**Risk factors**							
BMI, kg/m^2^, >25 (%)	33 (49)	82 (40)	0.23	104 (58)	208 (39)	0.001	0.5
Prior MI, Yes (%)	28 (41)	3 (1.5)	<0.001	31 (17)	5 (0.9)	<0.001	0.003
Known CHD (no prior MI), Yes (%)	6 (9)	6 (3)	0.05	30 (17)	23 (4)	<0.001	0.2
Hypertension, Yes (%)	34 (50)	34 (17)	<0.001	73 (41)	97 (18)	<0.001	0.4
Hypercholesterinemia, Yes (%)	22 (32)	61 (30)	0.7	96 (54)	109 (20)	<0.001	0.07
Diabetes, Yes (%)	2 (3)	6 (3)	1.0	11 (6)	3 (1)	<0.001	0.3
Smoking, Yes (%)	17 (25)	38 (19)	0.3	44 (25)	133 (25)	1.0	1.0
**Physical activity**							
Light to moderate physical activities							
>3 times per week, (%)	23 (34)	80 (39)	0.4	101 (56)	389 (72)	<0.01	0.06
High intensity exercise							
>1 time per week, (%)	3 (4)	30 (15)	0.04	35 (20)	112 (21)	0.7	0.009
Mountain sports activities							
>2 weeks per year, (%)	30 (44)	94 (46)	0.8	55 (31)	311 (58)	<0.001	0.2

Data are presented as means (±SD) for characteristics, frequencies (percentages) for risk factors and physical activity habits. *p*-values are calculated by the use of Chi-square or Fisher’s exact test. ^a^ indicates *p*-value for differences between skiers and hikers with SCD.

**Table 2:** Comparison of adjusted odds ratios (95% confidence intervals) with regard to risk factors and physical activity habits among SCD victims and Controls derived from logistic regression analyses.

Study Population	Skiers		Hikers	
	with SCD vs. Controls	*p*-Value	with SCD vs. Controls	*p*-Value
**Risk factors**				
BMI, kg/m^2^, >25 (%)	0.54 (0.25–1.17)	0.12	1.2 (0.8–1.8)	0.4
Prior MI, Yes (%)	92.8 (22.8–379.1)	<0.001	10.9 (3.8–30.9)	<0.001
Known CHD (no prior MI), Yes (%)	4.8 (1.1–21.2)	0.04	4.7 (2.4–9.2)	<0.001
Hypertension, Yes (%)	9.0 (4.0–20.6)	<0.001	1.5 (0.9–2.4)	0.1
Hypercholesterinemia, Yes (%)	0.59 (0.23–1.53)	0.28	3.4 (2.2–5.2)	<0.001
Diabetes, Yes (%)	1.1 (0.12–9.5)	0.94	7.4 (1.6–34.3)	<0.001
Smoking, Yes (%)	2.0 (0.78–4.9)	0.15	0.64 (0.4–1.1)	0.08
**Physical activity**				
Light to moderate physical activities				
>3 times per week, (%)	2.0 (0.82–4.6)	0.13	0.9 (0.6–1.4)	0.64
High intensity exercise				
>1 time per week, (%)	0.17 (0.04–0.74)	0.02	1.4 (0.8–2.3)	0.22
Mountain sports activities				
>2 weeks per year, (%)	1.2 (0.55–2.6)	0.65	0.23 (0.1–0.4)	<0.001

Abbreviations: SCD, sudden cardiac death; BMI, body mass index; MI, myocardial infarction; CHD, coronary heart disease.

## Data Availability

All data available in refs. [10,11].
